# The cortical hem lacks stem cell potential despite expressing SOX9 and HOPX

**DOI:** 10.1002/dneu.22899

**Published:** 2022-09-20

**Authors:** Alessia Caramello, Christophe Galichet, Miriam Llorian Sopena, Robin Lovell‐Badge, Karine Rizzoti

**Affiliations:** ^1^ Laboratory of Stem Cell Biology and Developmental Genetics The Francis Crick Institute London UK; ^2^ UK Dementia Research Institute Imperial College London London UK; ^3^ Bioinformatics and Biostatistics Science Technology Platform Francis Crick Institute London UK

**Keywords:** dentate gyrus, differentiation, gliogenesis, HOPX, SOX9, stem cell potential

## Abstract

The adult dentate gyrus (DG) of rodents hosts a neural stem cell (NSC) niche capable of generating new neurons throughout life. The embryonic origin and molecular mechanisms underlying formation of DG NSCs are still being investigated. We performed a bulk transcriptomic analysis on mouse developing archicortex conditionally deleted for *Sox9*, a SoxE transcription factor controlling both gliogenesis and NSC formation, and identified *Hopx*, a recently identified marker of both prospective adult DG NSCs and astrocytic progenitors, as being downregulated. We confirm SOX9 is required for HOPX expression in the embryonic archicortex. In particular, we found that both NSC markers are highly expressed in the cortical hem (CH), while only weakly in the adjacent dentate neuroepithelium (DNE), suggesting a potential CH embryonic origin for DG NSCs. However, we demonstrate both in vitro and in vivo that the embryonic CH, as well as its adult derivatives, lacks stem cell potential. Instead, deletion of *Sox9* in the DNE affects both HOPX expression and NSC formation in the adult DG. We conclude that HOPX expression in the CH is involved in astrocytic differentiation downstream of SOX9, which we previously showed regulates DG development by inducing formation of a CH‐derived astrocytic scaffold. Altogether, these results suggest that both proteins work in a dose‐dependent manner to drive either astrocytic differentiation in CH or NSC formation in DNE.

## 1 INTRODUCTION

In the adult brain of rodents, neural stem cells (NSCs) can be found in three restricted niches where physiological adult neurogenesis still occurs: the subventricular zone (SVZ) of the lateral ventricles, the subgranular zone (SGZ) of the dentate gyrus (DG), and the cells lining the third ventricle in the hypothalamus (Jurkowski et al., 2020). The balance between adult NSCs quiescent and active states is carefully controlled by several pathways to ensure long‐term maintenance of neuronal production (Urbán et al., 2019).

The *Cornus Ammonis* (CA) and DG, which together form the hippocampus, originate from two adjacent domains within the archicortex (ARK): the hippocampal neuroepithelium (HNE) and the dentate neuroepithelium (DNE), respectively (Morales and Mira, 2019; Urbán and Guillemot, 2014). DG progenitors are located within the DNE in early embryogenesis and, as development proceeds, they delaminate and migrate through the parenchyma along the dentate migratory stream to reach the forming DG where they differentiate into mature granule neurons (Morales and Mira, 2019; Urbán and Guillemot, 2014). Ventral to the DNE, the cortical hem (CH), later fimbrial epithelium (FE), contains progenitors first giving rise to Cajal–Retzius (CR) cells (Yoshida et al., 2006), then to astrocytes forming the fimbrial glial scaffold necessary for migration of DNE neuronal progenitors toward the DG (Caramello et al., 2021).

The embryonic origin of adult DG NSCs is still under debate (Morales and Mira, 2019): some have shown they are generated by a common precursor also giving rise to granule neurons (Berg et al., 2019); others suggest they originate from the ventral part of the hippocampus (Li et al., 2013). Single‐cell data showed that the transcriptome of DG neuronal precursors changes greatly as cells transition from embryonic to adult DG NSCs, particularly within the first 2 weeks of postnatal life (Hochgerner et al., 2018), suggesting that the early postnatal period may be important for establishment of the future adult NSC population. However, the expression of some genes is conserved in embryonic and adult NSCs. For example, *Homeodomain only protein X* (HOPX, also known as HOD‐1) is expressed in adult DG NSCs (Shin et al., 2015), where it is necessary to maintain their quiescence (Li et al., 2015). HOPX is also present in the embryonic DNE from E10.5 in mice (Berg et al., 2019) and gestational week 10 in humans (Zhong et al., 2020), where it is considered an early marker of adult DG NSCs. These observations suggest HOPX might be necessary at early stages of embryonic DG development for establishment of the adult DG NSC pool.

HOPX is also expressed in mature astrocytes, both in the dorsal brain parenchyma (Morel et al., 2017) and in the septal wall SVZ (Mizrak et al., 2019). Furthermore, it is present during astrocytic differentiation, both in adult SVZ NSCs biased to generate astrocytes (Zweifel et al., 2018) and in embryonic spinal cord progenitors, where its spatiotemporal expression profile matches that of astrocytic genes induced by SOX9 and NF1A (Sagner et al., 2021). In absence of these two factors, *Hopx* levels are reduced (Kang et al., 2012). Altogether, these findings suggest HOPX may also be involved in astrocytic differentiation; however, its requirement in this context has not been formally demonstrated.

Although the role of HOPX in DG development has recently become evident, its expression pattern during this process has not been thoroughly characterized. In both mouse (Berg et al., 2019) and human (Zhong et al., 2020) ARK, HOPX appears to be expressed both in the DNE and the underlying CH/FE (Mühlfriedel et al., 2005) raising the possibility that adult NSC progenitors may reside in either or both of these two domains. Alternatively, in the CH, HOPX might instead be involved in fimbrial glial scaffold differentiation.

The SRY‐related high‐mobility group box (SOX) transcription factor 9 (SOX9) is expressed in the murine neuroepithelium from E9.5 and is controlling both induction of stem cell fate and the progenitor switch to astrogliogenesis (Caramello et al., 2021; Kang et al., 2012; Scott et al., 2010; Stolt et al., 2003). To identify new regulatory mechanisms underlying adult DG NSC formation and clarify their regional origin, we performed bulk RNA sequencing (RNAseq) from E13.5 dissected ARK deleted for *Sox9* in complementary regions, as shown previously (Caramello et al., 2021), and identified *Hopx* as being significantly downregulated. We subsequently analyzed HOPX expression in vivo, in early and late ARK development, and showed that it is highly expressed in the CH/FE, while only weakly in the DNE. We confirm its expression depends on SOX9, both in embryonic CH and DNE progenitors, as previously shown in the developing spinal cord (Kang et al., 2012), as well as adult DG NSC. Because of the co‐expression and comparatively higher levels of SOX9 and HOPX in the CH, its potential to originate DG NSCs was then investigated in vitro using the neurosphere assay and in vivo by lineage tracing, using *Wnt3a^Cre^* to label CH‐derived cells (Yoshida et al., 2006). Both approaches show that CH cells do not have stem cell properties and are not the embryonic source of adult DG NSCs. Conversely, we show both HOPX expression and NSC formation and expansion in the adult DG require SOX9 in the DNE. Therefore, despite SOX9 and HOPX being present in both CH and DNE, their expression per se is not sufficient to define embryonic DG NSC potential. We instead propose that high levels of SOX9 and HOPX in the CH are most likely associated with astrocytic differentiation, suggesting that increasing levels of both factors along the DNE/CH neuroepithelium axis are associated with a transition from neural to astrocytic progenitor fate.

## 2 MATERIAL AND METHODS

### 2.1 Animals, husbandry, and genotyping

All animal experiments carried out were approved under the UK Animals (Scientific Procedures) Act 1986 and under the project licenses no. 70/8560 and PP8826065. Husbandry, breeding, ear biopsies, and vaginal plug (VP) checks were undertaken by the Biological Research Facility Team of the Francis Crick Institute. Mice were kept in individually ventilated cage with access to food and water ad libitum. The day of the VP was considered as embryonic day 0.5 (E0.5). All mouse lines used in this studies have been previously described: *Sox9^fl/fl^* conditional targeted mutation, MGI: 2429649 (Akiyama et al., 2002); *Sox1^Cre/+^* targeted mutation, MGI: 3807952 (Takashima et al., 2007); *Nestin‐Cre* transgenic mutation, MGI: 2176173 (Tronche et al., 1999); *Wnt3a^IRES‐Cre^* targeted mutation, MGI: 98956 (Yoshida et al., 2006); and *R26R^tomato^* targeted mutation, MGI: 3809523 (Madisen et al., 2010). Genotyping was performed using Transnetyx.

### 2.2 RNA extraction and sequencing

E13.5 archicortices were dissected out in sterile chilled phosphate‐buffered saline (PBS), snap frozen in liquid nitrogen, and stored at −80°C. RNA was extracted using RNeasy Plus Micro kit (Qiagen) and quantified with NanoDrop (Thermo Fisher Scientific). RNA quality control, cDNA library synthesis using KAPA mRNA HyperPrep Kit (Illumina), and sequencing, performed on HiSeq4000 (Illumina), were performed by the Genomic Park and Advanced Sequencing facilities of the Francis Crick Institute. Sequencing analysis was performed by Miriam Llorial, from the bioinformatics and biostatistics facility of the Francis Crick Institute. Read adaptor removal and quality trimming were carried out with Trimmomatic (version 0.36) (Bolger et al., 2014). Reads were then aligned to the mouse genome, using Ensembl GRCm38 ‐ release 89 as reference. Read alignment and gene level quantification were performed by STAR alignment (v.2.5.2a) (Dobin et al., 2013) together with RSEM package (v.1.2.31) (Li and Dewey, 2011). Differential expression analysis was carried out with DESEq2 (DESeq2_1.20.0) (Love et al., 2014) within R programming environment. Genes are called differentially expressed if *p*_adj_ < .05. RNAseq reads quality was assessed with the FastQC program, where multiple parameters are analyzed, such as millions of sequences obtained, percent of duplicate reads, percent of GC content, percent of mRNA or rRNA, and number of genes with at least five reads. All samples passed RNA quality control, with an average RNA integrity number (RIN) of 9.6, and sequencing quality control. After sequencing, each sample had between 20 and 30 million reads with a percentage of duplication of around 50%–60%. From each sample, around 20,000 genes were detected with at least five reads, of which on average 90% aligned with coding regions.

Lists of differentially expressed genes (DEGs) were generated for each comparison. DEGs were considered only when the *p*_adj_‐value was below .05. Lists of DEGs were manually analyzed to identify potentially interesting genes. For this analysis, only genes with a BaseMean above 100 were considered, as it would correct for very low expressed genes that would not have a biological effect even with a high fold change. Lists of DEGs were also used to perform pathway analysis with MetaCore.

### 2.3 Analysis of putative SOX9 transcription factor binding sites

Position frequency matrix for SOX9 was obtained from Jaspar2018 (Khan et al., 2018). The R package “PWMEnrich” (Stojnic & Diez, 2015) was used to obtain a Position Weith Matrix (PWM) and to find matches in 10 Kb upstream/downstream of *Hopx* gene (chr5: 77077073–77102813, UCSC Genome Browser on Mouse Dec. 2011(GRCm38/mm10) Assembly). Hits were saved as a .bed file and uploaded onto UCSC genome browser for visualization purposes. To further infer their functional activity, these domains were compared to chromatin state data generated from ChIP‐seq and ATAC‐seq analyses performed by ENCODE 3 (Sloan et al., 2016) and available on UCSC website. We specifically looked at (i) enhancers‐gene map, (ii) enhancer regions specifically associated with *Hopx* promoter, and (iii) chromatin states during forebrain development.

### 2.4 Neurosphere assays

Embryonic ARK or adult hippocampus was dissected and cultured in proliferation medium as described previously (Scott et al., 2010). For differentiation assays, neurospheres were placed on reduced growth factor Matrigel (BD biosciences) coated coverslips in differentiation medium (Scott et al., 2010). 7DIV‐differentiated neurospheres were then processed for staining.

### 2.5 Tissue harvesting and staining

Pregnant dams were killed by cervical dislocation, embryos were harvested, and heads were dissected out on chilled PBS. E13.5 whole heads and E18.5 dissected‐out brains were fixed by immersion in chilled 4% paraformaldehyde (PFA) at 4°C for 1–2 h. Heads/brains were then washed in PBS, cryopreserved in 30% sucrose, embedded in OCT, and kept at −80°C. Samples were sectioned at 14 μm using a cryostat (Leica), placed on Superfrost plus slides, and either stored at −80°C or immediately used for staining.

Adult mice were perfused intracardially with chilled 4% PFA and brains were dissected out and embedded in OCT. Samples were sectioned at 50 μm using a cryostat (Leica) and free‐floating sections were either cryopreserved and stored at −80°C or immediately used for staining.

Whole neurospheres and differentiated neurospheres were fixed with chilled 4% PFA for 20 min at 4°C. Samples were washed in PBS and stained.

For immunofluorescence, samples were incubated in blocking solution (10% donkey serum in 0.1% Triton X‐100 PBS) for at least 30 min and then incubated overnight at 4°C with primary antibodies diluted in blocking solution (Table 1). For some antibodies, antigen retrieval was performed prior to the blocking solution step by placing the samples in 10% target retrieval solution pH6.1 (Dako) diluted 1:10 in distilled water, for 30 min at 65°C or 15 min at 95°C (Table 1). The following day, samples were washed twice in 0.1% Triton X‐100 PBS for 5 min, incubated for 1 h at room temperature with secondary antibodies (Table 2), and DAPI diluted 1:500 in blocking solution in a dark humidified chamber. After two 5‐min washes in PBS, samples are air‐dried and mounted under coverslip with Aqua‐poly/Mount (Park Scientific).

**TABLE 1:** List of primary antibodies used

Antigen	Dilution	Host	Vendor	Catalog #	65^o^C	95^o^C
LEF1	1:200	Rabbit	Cell Signalling	2230P	e	e
GFAP	1:500	Mouse	Sigma	C9205		
GLAST	1:200	Guinea pig	Millipore	AB1782		e
HOPX	1:100	Rabbit	Proteintech	11419‐1‐AP		e
Ki67	1:200	Rabbit	Abcam	ab16667	e	
PDGFRa	1:200	Goat	R&D systems	AF1062		
PROX1	1:500	Rabbit	BioLegend	PRB‐238C	e	a
REELIN	1:200	Mouse	Abcam	Ab78540		
SOX2	1:500	Goat	Neuromics	GT15098		
SOX9	1:200	Goat	R&D system	AF3075	e	e

*Note*: When necessary, antigen retrieval protocol (30 min in 65°C water bath or 15 min in 95°C decloaking chamber) was performed for the indicated samples (e: embryos; a: adults).

**TABLE 2:** List of secondary antibodies and nuclear staining used

Fluorophore	Host/reactivity species	Vendor	Catalog #
Alexa 568	Donkey anti‐rabbit	Thermo Fisher Scientific	A10042
Alexa 647	Donkey anti‐rabbit	Thermo Fisher Scientific	A31573
Alexa 568	Donkey anti‐goat	Thermo Fisher Scientific	A11057
Alexa 647	Donkey anti‐goat	Thermo Fisher Scientific	A21447
Alexa 594	Donkey anti‐mouse	Thermo Fisher Scientific	A21203
Alexa 555	Donkey anti‐mouse	Thermo Fisher Scientific	A31570
Alexa 647	Donkey anti‐guinea pig	Thermo Fisher Scientific	A21450
DAPI 300 μM		Thermo Fisher Scientific	D1306

### 2.6 Image processing and statistical analysis

Immunofluorescence stainings were imaged using a Leica TCS SPE confocal microscope with 10×, 20×, and 40× objectives. LAS AF software was used for acquisition. Acquisitions were performed as 1.5 μm Z‐stacks, with bidirectional X. Fiji was used for image analysis, cell counting, and pixels per area quantification. QuPath was used for cell counting in Figure 5. All quantifications were performed on three to five different images per analyzed area for each sample.

Quantification of HOPX expression as pixels per area was performed in Fiji by first defining a threshold of HOPX signal intensity (below threshold is background, above threshold is real signal), then drawing a region of interest (ROI) around the area of interest and quantifying the percentage of pixels within the ROI that bear a HOPX signal intensity above the defined threshold. Finally, by multiplying the percentage of pixels above threshold by total number of pixels of the ROI, we obtained the number of pixels where HOPX expression is above threshold (HOPX+ pixels/area).

Statistical analysis was performed on Prism 9 (Graphpad), calculating student's two‐sided unpaired *t*‐tests, when comparing two groups, or ordinary one‐way ANOVA, when comparing one variable in three groups. When performing ANOVA, multiple comparison between each experimental group was then performed with Tukey's test. Analyses were performed parametrically, upon confirmation that majority of samples in the analysis had normal distribution (performed on Prism). Full details of statistical analyses can be found in the source data.

Histograms represent average quantification from the indicated number of biological replicates (*n*). Error bars for cell number quantification represent standard deviation (SD). Data shown as percentage were processed with angular transformation before statistical analysis; *p*‐value is indicated as follows: ns, *p* > .05; *p ≤ .05; **p ≤ .01; ***p ≤ .001; ****p ≤ .0001.

**FIGURE 1:**
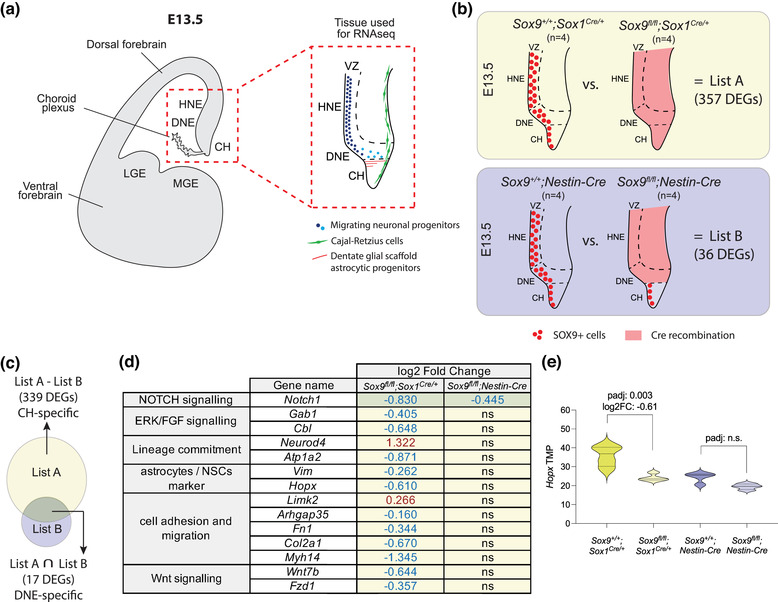
Comparative bulk transcriptomic analysis of embryonic dissected ARK. (a) Schematic of a coronal section of a developing mouse forebrain at E13.5 with red‐dashed square indicating the ARK, the region of the embryonic brain dissected and used for bulk RNAseq. On the right, zoomed detailed description of the neuronal progenitors initially present in the DNE/HNE (dark blue dots) then migrating within the parenchyma (light blue dots). Below the DNE, the CH is the source of Cajal–Retzius cells (in green) and of glial scaffold astrocytic progenitors (in red). (b) Schematic of SOX9 expression (red dots) and Cre recombination pattern (red shade) in E13.5 *Sox9^fl/fl^;Sox1^Cre/+^* and *Sox9^fl/fl^;Nestin‐Cre* ARK compared to their controls. DEGs identified in *Sox9^fl/fl^;Sox1^Cre/+^* mutants compared to their controls are indicated as List A, while DEGs identified in *Sox9^fl/fl^;Nestin‐Cre* mutants compared to their controls are indicated as List B. (c–e) Lists A and B were compared to identify DEGs potentially specific to the CH and DNE. Based on Cre activity pattern, DEGs identified in *Sox9^fl/fl^;Sox1^Cre/+^* mutants (List A) but not in *Sox9^fl/fl^;Nestin‐Cre* mutants (List B) are more likely to be specific of CH only (List A – List B; 339 DEGs), while DEGs identified in both *Sox9^fl/fl^;Sox1^Cre/+^* (List A) and *Sox9^fl/fl^;Nestin‐Cre* (List B) mutants are more likely to be DNE specific (List A  ∩ List B: 17 DEGs) (c). A highlight of DEGs shortlisted with this method is shown in panel (d). Yellow and green shades indicate whether each gene is DNE or CH specific, respectively, based on the comparison described above. (e) *Hopx* TMP showing its expression is significantly downregulated in *Sox9^fl/fl^;Sox1^Cre/+^* mutants compared to controls, suggesting it might be expressed in the CH. LGE, lateral ganglionic eminence; MGE, medial ganglionic eminence; ARK, archicortex; VZ, ventricular zone; HNE, hippocampal neuroepithelium; DNE, dentate neuroepithelium; CH, cortical hem; DEG, differentially expressed genes; PCA, principal component analysis; ns, nonsignificant; TPM, transcripts per million; FC, fold change

**FIGURE 2:**
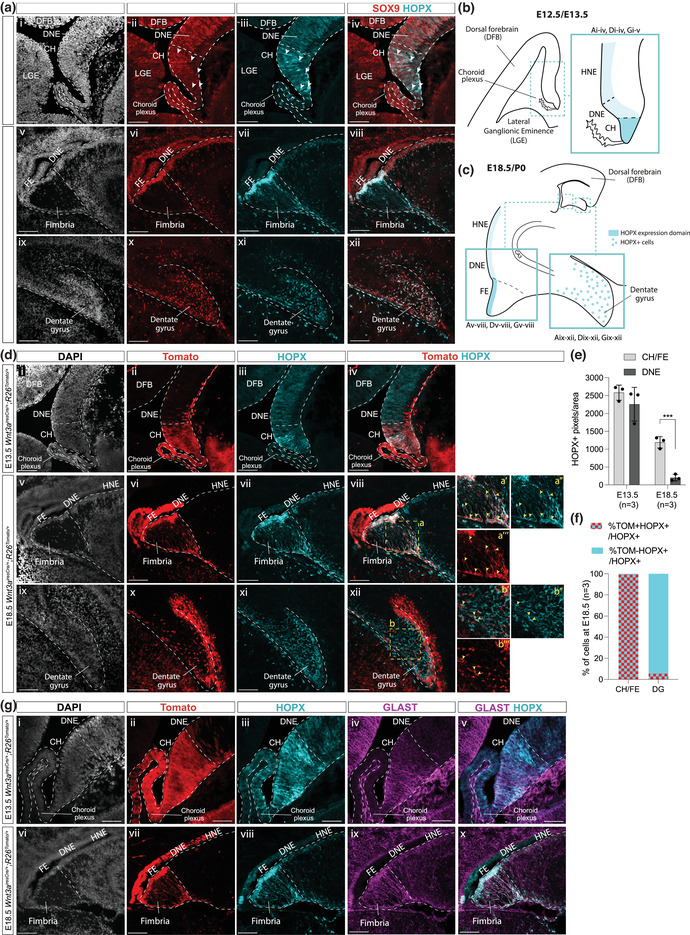
Characterization of HOPX expression within the developing ARK, compared to SOX9 and astrocytic progenitors’ markers. (a) Immunostaining for HOPX and SOX9 on *Sox9^fl/fl^* within the developing ARK at E12.5 (i–iv) and at E18.5 (v–viii) and in the forming DG at E18.5 (ix–xii). SOX9+;HOPX+ double positive cells at E12.5 (i–iv) are indicated with the white arrowhead. (b, c) Schematic representation of regions of the developing archicortex at E12.5/E13.5 (b) and developing dentate gyrus (c, right) and FE (c, left) analyzed in panels (a), (d), and (g), with an indication of HOPX level of expression in different regions/cells. (d) Immunostaining for HOPX and Tomato on *Wnt3a^iresCre/+;^R26^Tomato/+^* E13.5 (i–iv) and E18.5 (v–xii) embryos. The area indicated by the yellow‐dashed squares (a, b) is shown at higher magnification on the right (aʹ–a‴ and bʹ–b‴). Yellow and white arrowheads indicate HOPX+;Tomato+ and HOPX+;Tomato– cells, respectively. (e, f) Analysis of HOPX expression in the developing ARK quantified as HOPX+ pixels/area at E13.5 and E18.5 in CH/FE versus DNE (e) showing HOPX is significantly more expressed in the CH/FE (1190.57 ± 159.62) compared to the DNE (202.35 ± 91.12, *t*‐test *p* = .0007) at E18.5; percentage of Tomato+ and Tomato– HOPX+ cells on total HOPX+ cells population in the CH/FE and forming DG at E18.5 (f). (g) Triple immunostaining for HOPX, Tomato, and GLAST on E13.5 (i–v) and E18.5 (vi–x) developing ARK of *Wnt3a^iresCre/+;^R26^Tomato/+^* embryos, showing HOPX expression overlaps, at least in part, with GLAST+ astrocytic progenitors in the CH and FE/fimbria. ARK, archicortex; DNE, dentate neuroepithelium; HNE, hippocampal neuroepithelium; CH, cortical hem; DFB, dorsal forebrain; LGE, lateral ganglionic eminence; CP, choroid plexus; FE, fimbrial epithelium; DG, dentate gyrus. Scale bars represent 50 μm in (a [i–iv]) and (g [i–v]), and 100 μm in (a [v–xii]), (d), and (G [vi–x]).

Images in Figures 4a and 5a were created with BioRender.com, while cartoons in Figures 1a,b and 2b,c were created with Adobe Illustrator.

## 3 RESULTS

### 3.1 Transcriptomic analysis of the E13.5 developing ARK upon differential conditional deletion of *Sox9*

We used differential conditional deletion approaches to ablate *Sox9* expression in complementary regions of the developing ARK, as previously described (Caramello et al., 2021). We then performed bulk RNAseq of dissected ARK (Figure 1a) from quadruplicate E13.5 *Sox9^fl/fl^;Sox1^Cre/+^* and *Sox9^fl/fl^;Nestin‐Cre* mutants compared to *Sox9^+/+^;Sox1^Cre/+^* and *Sox9^+/+^;Nestin‐Cre* controls, respectively (Figure 1b). *Sox1^Cre^* is active in the whole neural plate from E8.5 (Takashima et al., 2007), while *Nestin‐*Cre is active from E11.5 in the ventral forebrain, reaching the DNE by E13.5, and mostly inactive in the adjacent CH (Figure 1b) (Tronche et al., 1999). Therefore, comparisons of DEGs between these mutants should identify and distinguish downstream targets of SOX9 preferentially expressed in the CH and the DNE.

Sequenced samples were first analyzed by principal component analysis (PCA) (Figure S1a,b) and genotypes confirmed by examining *Sox9* expression (Figure S1c). While deletion of *Sox9* underlined variance in *Sox9^fl/fl^;Sox1^Cre/+^* samples compared to controls, it did not in *Sox9^fl/fl^;Nestin‐Cre* versus controls (Figure S1a,b). In consequence, we identified 357 differentially expressed genes (DEGs) in *Sox9^fl/fl^;Sox1^Cre/+^* mutants compared to their controls (List A: 157 upregulated and 200 downregulated—Table S1), while only 36 DEGs were identified in *Sox9^fl/fl^;Nestin‐Cre* mutants compared to their controls (List B: 11 upregulated and 25 downregulated—Table S2). Consistent with *Sox1^Cre/+^* and *Nestin‐Cre* activity patterns, this result indicates that early DG development is significantly more affected in *Sox9^fl/fl^;Sox1^Cre/+^* compared to *Sox9^fl/fl^;Nestin‐Cre* animals (Figure 1). The lower number of DEGs found in the latter compared to their controls might also reflect the delayed activity of *Nestin‐Cre* compared to *Sox1‐Cre* in the DNE, which allows for transient expression of *Sox9* before it is deleted (Caramello et al., 2021).

We then compared DEGs found in *Sox9^fl/fl^;Sox1^Cre^* mutants (List A) with those found in *Sox9^fl/fl^;Nestin‐Cre* mutants (List B) with the aim of dissecting the role of SOX9 in subregions of the ARK (Figure 1c). DEGs found in *Sox9^fl/fl^;Sox1^Cre/+^* but not in *Sox9^fl/fl^;Nestin‐Cre* mutants should specifically indicate genes regulated by SOX9 in the CH (List A minus [–] List B; Table S3). Conversely, genes commonly affected in both mutants should focus on pathways regulated by SOX9 in the DNE (List A intersection [∩] List B; Table S4). We screened these lists to identify pathways in which SOX9 is already known to be involved or that are relevant to this developmental context (Figure 1d). We identified the neurogenic factor *NeuroD4* to be upregulated in *Sox9^fl/fl^;Sox1^Cre/+^* mutants compared to their controls, and conversely the astrocytic markers (*Atp1a2*, *Vim*) to be downregulated, potentially indicative of the role of *Sox9* in gliogenesis induction, which, in its absence, is delayed in favor of neurogenesis (Kang et al., 2012; Martini et al., 2013). We also observed a reduction in components of the NOTCH and WNT pathways (*Notch1*, *Wnt7b*, *Fzd1*), and of genes involved in cell adhesion, migration, and epithelial to mesenchymal transition (EMT; *Limk2*, *Arhgap35*, *Fn1*, *Col2a1*, *Myh14*). These results are consistent with SOX9 being involved in or interacting with both NOTCH and WNT pathways (Leung et al., 2016; Martini et al., 2013), and with previously identified roles of SOX9 in gliogenesis induction (Kang et al., 2012) as well as EMT during neural crest cell development (Lincoln et al., 2007; Liu et al., 2013). We also identified markers specific to NSCs and astrocytic progenitors, such as *Vimentin*, encoding for an intermediate filament (Pankratz et al., 2007), and *Hopx*, encoding for the HOPX/HOD‐1 transcription factor (Berg et al., 2019; Mizrak et al., 2019; Morel et al., 2017). *Hopx* particularly piqued our attention, because it has been recently identified as being a marker of adult DG NSCs, but one that is expressed in the relevant region of the CNS during embryonic development (Berg et al., 2019). Moreover, from a UCSC Genome Browser analysis, we could find several predicted SOX9 transcription binding sites (TBSs) upstream of the *Hopx* transcription starting site (Figure S2), suggesting indeed SOX9 could directly control HOPX expression. We first proceeded with validation of our transcriptomic data by analyzing HOPX expression pattern in the developing ARK.

### 3.2 HOPX is highly expressed in CH/FE compared to DNE and is regulated by SOX9 in both domains

We first analyzed HOPX expression at different stages of DG development, comparing it to SOX9 and using *Wnt3a^iresCre/+^;R26^Tomato/+^* (Yoshida et al., 2006) to demarcate DNE and CH. In agreement with previous studies (Ota et al., 2018), HOPX expression was found to be both nuclear and cytosolic; therefore, we quantified its expression level as HOPX+ pixels per relevant area. At E12.5, HOPX is weakly expressed in the DNE, while toward the most ventral part of the ARK, its expression intensifies in a few cells (Figure 2a [i–iv]), most of which are also expressing SOX9 (71.7% ± 3.61%). This region likely corresponds to the CH, as shown by immunostaining for HOPX on E13.5 *Wnt3a^iresCre/+^;R26^Tomato/+^* embryos (Figure 2d [i–iv]). Despite few cells in the CH highly expressing HOPX, we did not identify a significant increase in HOPX expression, quantified as HOPX+ pixels per relevant area, in the CH compared to the DNE at this stage (Figure 2e). Later, at E18.5, HOPX is still weakly expressed in the DNE and the overlying hippocampal neuroepithelium (HNE; Figure 2a [v–viii]), which corresponds to the *Wnt3a^iresCre/+^;R26^Tomato/+^* Tomato– domain (Figure 2d [v–viii]), while its expression is significantly higher in the Tomato+ fimbrial epithelium (FE; Figure 2d [v–viii], quantified in Figure 2e) where SOX9 also seems expressed at higher levels (Figure 2a [v–viii]). We observed HOPX+ signal in nuclei, and in Tomato*+* fibers extending from the FE (Figure 2d [v–viii], magnified in aʹ–a‴). These correspond to the fimbrial glial scaffold, because both Tomato and HOPX signal overlap with GLAST expression both at E13.5 and E18.5 (Figure 2g). At E18.5, HOPX is also expressed in SOX9+ cells migrating toward and within the forming DG (Figure 2a [ix–xii]). However, compared to the FE, where most HOPX+ cells are Tomato+ (Figure 2f), in the forming DG most HOPX+ cells are not expressing Tomato in *Wnt3a^iresCre/+^;R26^Tomato/+^* embryos (Figure 2d [ix–xii],f), suggesting they do not originate from the CH/FE. We hypothesize that the few HOPX+;Tomato+ cells observed in the DG (Figure 2d [ix–xii]) represent ectopic activity, both temporal and spatial, of *Wnt3a^iresCre^* in the DNE (Figure 2d [iv]), as observed previously (Caramello et al., 2021). Taken together, these results indicate that during DG development, levels of expression of HOPX differ between regions within the ARK and correlate with that of SOX9.

**FIGURE 3:**
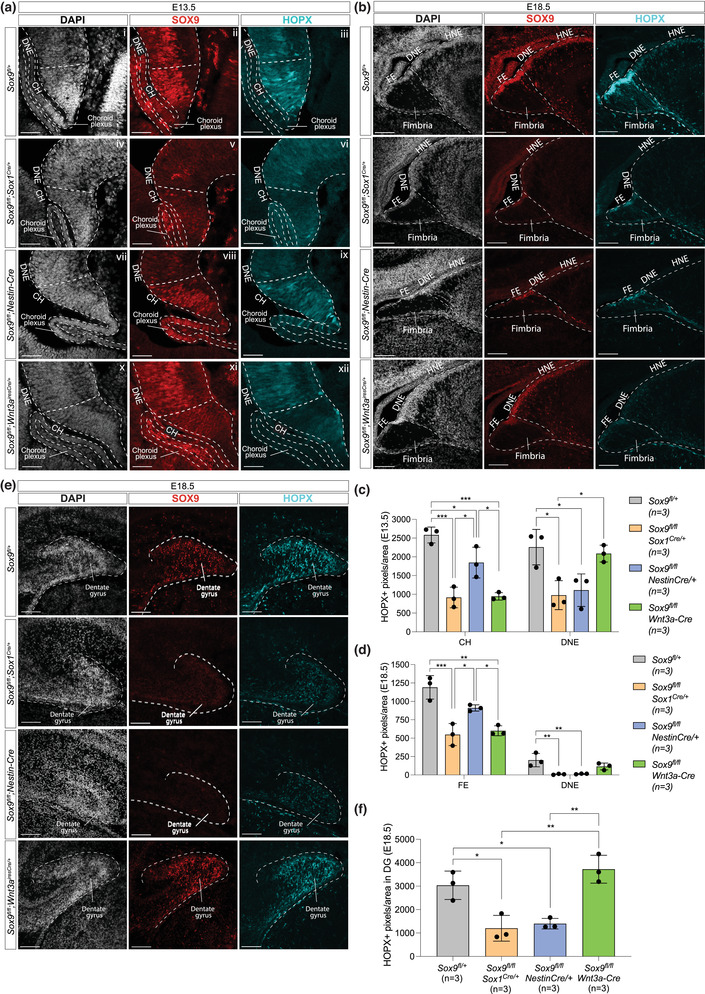
HOPX expression in the CH/FE, DNE, and forming DG is reduced upon *Sox9* deletion. (a–f) Double immunostaining for SOX9 and HOPX in developing ARK at E13.5 (a) and E18.5 (b) and in forming DG at E18.5 (e) from *Sox9^fl/fl^* control and *Sox9^fl/fl^;Sox1^Cre/+^*, *Sox9^fl/fl^;Nestin‐Cre*, and *Sox9^fl/fl^;Wnt3a^iresCre/+^* mutant embryos. HOPX expression in the CH at both ages (c, d) is significantly lower in *Sox9^fl/fl^;Sox1^Cre/+^* (E13.5: 915.80 ± 274.56, *p* = .0003; E18.5: 548.48 ± 149.24, *p* = .0007) and *Sox9^fl/fl^;Wnt3a^iresCre/+^* mutants (E13.5: 945.58 ± 99.06, *p* = .0004; E18.5: 600.48 ± 67.94, *p* = .0012) compared to controls (E13.5: 2583.37 ± 211.50; E18.5: 1190.57 ± 159.62), while in *Sox9^fl/fl^;Nestin‐Cre* mutants it is significantly higher compared to the other two mutants (E13.5: 1847.17 ± 410.30, *p* = .0132 and .0157; E18.5: 910.29 ± 42.52, *p* = .0216 and .0461; one‐way ANOVA *p* = .0002 for E13.5 and *p* = .0005 for E18.5 analyses). Conversely, HOPX expression is significantly lower in the DNE of *Sox9^fl/fl^;Sox1^Cre/+^* (E13.5: 977.23 ± 387.22, *p* = .0163; E18.5: 8.74 ± 6.73, *p* = .0074) and *Sox9^fl/fl^;Nestin‐Cre* mutants (E13.5: 1110.21 ± 434.03, *p* = .0288; E18.5: 14.88 ± 4.71, *p* = .0089) compared to controls (E13.5: 2259.38 ± 472.21; E18.5: 202.35 ± 91.12), while unaffected in *Sox9^fl/fl^;Wnt3a^iresCre/+^* mutants (one‐way ANOVA *p* = .0072 for E13.5 and *p* = .0049 for E18.5 analyses). Both analyses suggest HOPX expression in the CH and DNE relies on SOX9. Similarly, HOPX expression in the forming DG at E18.5 (f) is significantly reduced in *Sox9^fl/fl^;Sox1^Cre/+^* (1202.02 ± 548.69, *p* = .0108) and *Sox9^fl/fl^;Nestin‐Cre* mutants (1398.58 ± 225.49, *p* = .02) compared to controls (3036.37 ± 606.83), while not affected in *Sox9^fl/fl^;Wnt3a^iresCre/+^* mutants (one‐way ANOVA *p* = .0008), suggesting HOPX expression in the forming DG relies on SOX9 expression in the DNE. A schematic representation of the ARK, FE, and DG analyzed in this figure are shown in Figure 2b,c. ARK, archicortex; DNE, dentate neuroepithelium; HNE, hippocampal neuroepithelium; CH, cortical hem; FE, fimbrial epithelium; DG, dentate gyrus. Scale bars represent 50 μm in (a) and 100 μm in (b) and (e).

Then, to test whether SOX9 is regulating *Hopx*, as suggested by our RNAseq data, we analyzed HOPX expression levels. We examined E13.5 and E18.5 embryos with different patterns of *Sox9* deletion, using *Sox1^Cre/+^* for deleting the whole ARK, *Nestin‐Cre* for the DNE only, and *Wnt3a^iresCre/+^* for the CH only and observed a reduction of HOPX expression matching *Sox9* deletion patterns. More precisely, at E13.5, HOPX expression in the CH is significantly reduced in both *Sox9^fl/fl^;Sox1^Cre/+^* and *Sox9^fl/fl^;Wnt3a^iresCre/+^* mutants compared to controls (Figure 3a,c). In *Sox1^Cre^* mutants, HOPX is also reduced in the DNE, to a similar extent to that observed in *Sox9^fl/fl^;Nestin‐Cre* embryos. In the latter, HOPX expression levels are also reduced in CH, but to a lesser extent than in *Sox1^Cre^* and *Wnt3a^iresCre^* mutants, suggesting regulatory interactions between SOX9 and HOPX in both domains. Reduction of HOPX expression levels in the CH of *Sox9^fl/fl^;Sox1^Cre/+^* and *Sox9^fl/fl^;Wnt3a^iresCre/+^* mutants is not associated with a reduced cell expansion in this region (Figure S3), suggesting a reduction of HOPX levels rather than a loss in HOPX+ cells. The correlation between reduction of HOPX expression upon loss of *Sox9* is further confirmed in both DNE and FE at E18.5 (Figure 3b,d). These results support our findings from the transcriptomic analysis of *Sox9*‐deleted developing ARK, because they show that HOPX is mostly expressed in the CH and dependent on SOX9. Furthermore, they reveal that HOPX expression, despite being lower than in the CH, is also relying on SOX9 in the DNE.

Finally, HOPX is also expressed in the forming DG (Figure 2a [ix–xii]). We thus examined whether it similarly requires SOX9 in this domain. We found that HOPX expression in the forming DG at E18.5 is significantly reduced in *Sox9^fl/fl^;Sox1^Cre/+^* and *Sox9^fl/fl^;Nestin‐Cre* mutants (Figure 3e,f). This is not observed in *Sox9^fl/fl^;Wnt3a^iresCre/+^* mutants, in agreement with SOX9 being still present in the DG in these (Figure 3e [xi]). Furthermore, in both *Sox9^fl/fl^;Sox1^Cre/+^* and *Sox9^fl/fl^;Nestin‐Cre* mutants the proliferation rate of HOPX+ cells is significantly reduced in the E18.5 developing DG (Figure S4). Altogether, these results suggest that the continuous expression of HOPX in progenitor cells, from those in the DNE to DG, as well as their proliferation rate, relies on SOX9.

**FIGURE 4:**
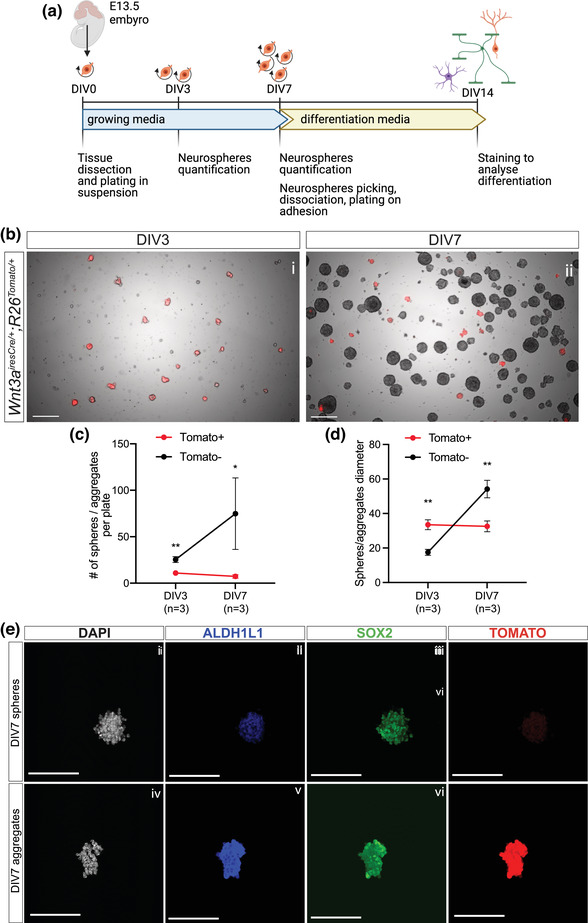
CH‐derived cells maintain their cell identity in vitro but do not have stem cell potential. (a) Schematic of neurospheres and differentiation assay from E13.5 *Wnt3a^iresCre/+;^R26^Tomato/+^* dissected embryonic ARK. (b–d) Representative pictures of neurospheres at DIV3 and DIV7 (c) and quantification of their number (d) and diameter (quantified in pixels; e) at both stages. The total number of Tomato– spheres is significantly higher both at DIV3 (25.47 ± 3.10, *t*‐test *p* = .0034) and increases significantly by DIV7 (74.82 ± 38.55, *t*‐test *p* = .0387), compared to Tomato+ aggregates, size of which remains the same over time (DIV3: 10.93 ± 2.61; DIV7: 7.27 ± 1.97). Conversely, at DIV3, Tomato+ aggregates are bigger (33.50 ± 2.89, *t*‐test *p* = .0012) than Tomato– spheres (17.50 ± 1.71), while at DIV7, the latter are significantly larger (54.17 ± 5.06, *t*‐test *p* = .0033) than Tomato+ aggregates (32.58 ± 3.14). (e) Immunostaining for ALDH1L1 and SOX2 on DIV7 Tomato– spheres and Tomato+ aggregates, showing DNE‐ and CH‐derived cells maintain their identity in vitro. CH, cortical hem; ARK, archicortex; DIV, days in vitro. Scale bars represent 200 μm in (b) and 100 μm in (e).

In conclusion, our data show that along the DNE/CH axis, HOPX expression both correlates with and relies on that of SOX9 in both domains. This suggests SOX9 is directly or indirectly regulating HOPX expression during ARK development, as shown previously in the developing spinal cord (Kang et al., 2012). Finally, we demonstrate that HOPX+ cells present in the forming DG at E18.5 do not originate from the CH and are thus likely of DNE origin. Instead in the CH/FE, co‐expression of GLAST and HOPX suggests these cells are differentiating toward a gliogenic lineage. However, because SOX9 is a general marker of embryonic NSCs (Scott et al., 2010) and HOPX is expressed in embryonic and adult DG NSCs (Berg et al., 2019; Li et al., 2015), co‐expression of both proteins at high levels in the CH/FE suggests this region could also be a source of adult DG NSCs. To investigate this possibility, we tested the embryonic CH stem cell potential in vitro.

### 3.3 iThe cortical hem maintains its cell fate potential in vitro

To test the stem cell potential of the CH, we performed an in vitro neurosphere and differentiation assay from embryonic ARK (Figure 4a). To distinguish CH‐ from DNE‐derived cells, we used dissociated ARK from E13.5 *Wnt3a^iresCre/+^;R26^Tomato/+^* embryos (as previously done for the RNAseq experiment; Figure 1a). Tissues were dissociated to single cells and cultured up to 7 days (Figure 4a). To monitor their proliferation capacity, which is indicative of stem cell potential, neurosphere numbers and size were quantified at 3 and 7 days (DIV; Figure 4b). While Tomato– neurospheres were readily obtained, Tomato+ cells seem to be only able to form uneven and mosaic aggregates in culture (Figure 4b). Furthermore, from DIV3 to DIV7, Tomato– DNE‐derived spheres grew in number and size, as expected, but Tomato+ CH‐derived aggregates did not expand and were similar to DIV3 (Figure 4c,d). Therefore, this assay shows CH‐derived cells do not have the potential to form neurospheres in vitro.

**FIGURE 5:**
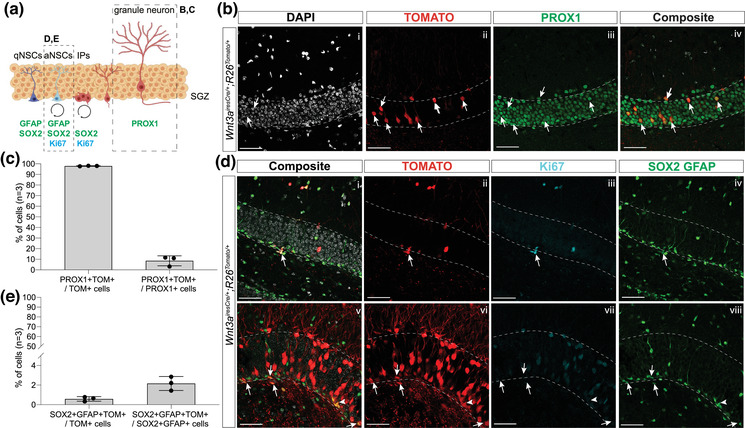
Few CH‐derived cells populate the adult DG. (a) Schematic of NSCs in the SGZ of the adult DG, with analyzed populations (GFAP+;SOX2+ NSCs and fully mature PROX1+ granule neurons) indicated by dashed squares. (b–e) Analysis of CH‐derived cells in adult DG in vivo with immunostaining for PROX1 (b) and Ki67, SOX2 and GFAP (d) together with endogenous Tomato to analyze CH‐derived cell fate toward NSCs and mature granule neurons, respectively, in the adult DG in 1‐month‐old *Wnt3a^iresCre/+;^R26^Tomato/+^* mice. (c, e) Quantification of percentage of Tomato+ NSCs and granule neurons on total Tomato+ cells shows the vast majority of Tomato+ cells in the adult DG are granule neurons. However, a small percentage of the total population of NSCs and granule neurons are Tomato+ (double positive cells are indicated by white arrows). CH, cortical hem; DG, dentate gyrus; SGZ, subgranular zone; NSCs, neural stem cells; qNSCs, quiescent NSCs; aNSCs, activated NSCs; Ips, intermediate progenitors. Scale bar represents 50 μm.

We then examined the identity of the Tomato+ cells in the aggregates and observed expression of the astrocyte progenitor marker ALDH1L1 (Figure 4e), which is normally expressed in CH from E13.5 (Caramello et al., 2021). This suggests that CH‐derived cells maintain their astrocytic fate in vitro. To further test whether CH‐ and DNE‐derived cells retain their differentiation potential in vitro, we manually sorted Tomato+ aggregates and Tomato– spheres and analyzed their fate in differentiating conditions (Figure 4a). In vivo, CH‐derived cells only give rise to CR cells and astrocytes of the glial scaffold, but not oligodendrocytes or granule neurons (Caramello et al., 2021; Gu et al., 2011; Liu et al., 2018). Conversely, DNE progenitors give rise to all cell types mentioned above except CR cells (Bielle et al., 2005). Upon in vitro differentiation, 42.46% ± 10.96% of cells were Tomato+ from aggregates, while almost no Tomato+ cells (0.07% ± 0.12%) were detected in differentiated Tomato– spheres (Figure 5), confirming that Tomato+ cells, predominantly CH derived, were enriched in aggregates (we cannot exclude a small contribution from DNE where Cre activity is observed at low levels; Figure 2d [iv]). Despite different starting origins, we did not observe any difference in the overall differentiation toward astrocytic (GFAP+), oligodendrocytic (PDGFRa+), or neuronal (PROX1+) fates when comparing sphere‐ and aggregate‐derived differentiated cells (Figure S5). Furthermore, Tomato+ cells from aggregates did not preferentially give rise to astrocytes (Figure S5) as they do in vivo. Conversely, Reelin+ cells differentiated exclusively from Tomato+ cells within Tomato– spheres (Figure 5b [v–viii]). This result was observed in two out of four samples analyzed, where some rare Tomato+ cells were present in Tomato– spheres (out of all DAPI+ cells identified, 0.32% and 0.46% were Tomato+ in these samples, respectively, compared to none in the other two; all these Tomato+ cells were Reelin+). These results suggest that the CR potential of CH‐derived cells can be maintained in vitro in presence of cues mimicking the in vivo situation, where they are surrounded by DNE‐derived cells. In conclusion, our results show that Tomato+ CH‐derived cells can retain some of their differentiation potential in specific conditions, but are unable to proliferate and form neurospheres in vitro, suggesting that at E13.5 the CH does not have NSC potential. We next examined whether such a potential may be acquired later by CH‐derived cells.

### 3.4 Adult DG NSCs do not originate from the embryonic CH

To test whether adult DG NSCs are generated at later stages of CH/fimbrial epithelium development, we performed a lineage tracing experiment on 1‐month‐old *Wnt3a^iresCre/+;^R26^Tomato/+^* mice. We found a few Tomato+ cells within the DG with a distribution skewed toward the junction of the two DG blades (Figure S6). We analyzed the fate of CH‐derived progenitors in the adult DG, staining for PROX1 for mature granule neurons and SOX2/GFAP for NSCs (Figure 5a). The vast majority of Tomato+ cells are PROX1+ granule neurons (98.23% ± 0.578%; Figure 5b,c), but these represent only a small proportion of granule neurons since only 8.62% ± 4.704% of PROX1+ cells are Tomato+ (Figure 5b,c). Although we cannot rule out the possibility that a few granule neurons might have been generated from CH‐derived cells, these results are most likely due to the ectopic activity of *Wnt3a^iresCre^* in the DNE during embryonic development, notably because we had previously found that a similar proportion of PROX1+ granule neurons (around 6.5%) are also recombined by *Wnt3a^iresCre^* in P2 pups (Caramello et al., 2021). Finally, the remaining 2.16% ± 0.708% of all Tomato+ cells in the adult DG are SOX2+;GFAP+ NSCs (Figure 5d,e), which represent a small fraction of the whole SOX2+;GFAP+ NSCs population (0.58% ± 0.228%; Figure 5d,e). Together, these data further suggest the CH is unlikely to be the source of adult DG NSCs.

**FIGURE 6:**
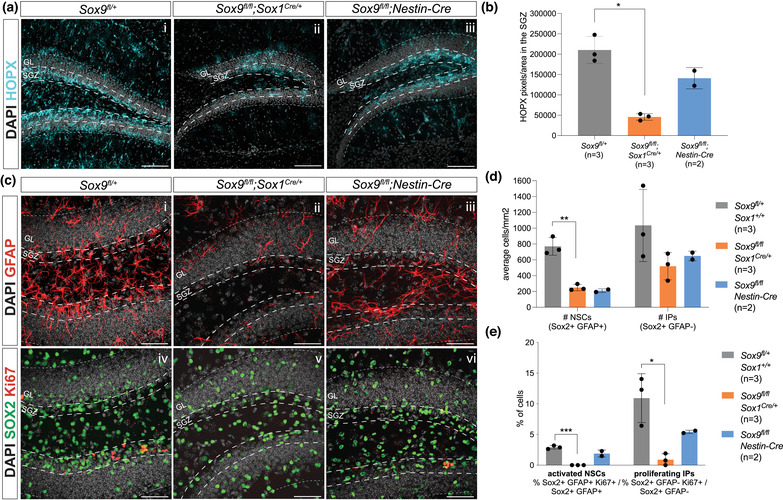
Pattern of embryonic *Sox9* deletion differentially affects NSCs in the adult DG. Immunostaining for HOPX in the adult DG of 2‐month‐old *Sox9^fl/fl^* control, *Sox9^fl/fl^;Sox1^Cre/+^*, and *Sox9^fl/fl^;Nestin‐Cre* mutant mice (a). HOPX expression levels in the SGZ (b, quantified as HOPX+ pixels/area, indicated by the long dashed line in panel a) were significantly lower in *Sox9^fl/fl^;Sox1^Cre/+^* mutants (45,710.73 ± 8082.16, *t*‐test *p* = .0011) compared to controls (210,408.44 ± 33,202.07). (c–e) Immunostaining for GFAP, SOX2, and Ki67 on 2‐month‐old *Sox9^fl/fl^* control, *Sox9^fl/fl^;Sox1^Cre/+^*, and *Sox9^fl/fl^;Nestin‐Cre* mutant mice DG to analyze total number and proliferation activity of NSCs and IPs in the SGZ (c; region quantified is indicated by the long dashed line). The total number of NSCs per square millimeter is significantly reduced in *Sox9^fl/fl^;Sox1^Cre/+^* mutants (249.31 ± 40.12, *t*‐test *p* = .0016) compared to controls (769.50 ± 111.02) (d). Proliferative activity of both NSCs and IPs is also significantly reduced in *Sox9^fl/fl^;Sox1^Cre/+^* mutants (NSCs: 0.00% ± 0.00%, *t*‐test *p* ≤ .0001; IPs: 0.89% ± 0.96%, *t*‐test *p* = .0102) compared to controls (NSCs: 2.91% ± 0.28%; IPs: 10.92% ± 3.99%) (e). Statistical analysis was not possible with *Sox9^fl/fl^;Nestin‐Cre* mutant, due to the limited number of samples available. SGZ, subgranular zone; GL, granular layer. Scale bars represent 100 μm in (a) and 50 μm in (c).

### 3.5 Embryonic *Sox9* deletion in the DNE affects HOPX expression and NSC formation in the adult DG

Finally, we tested whether embryonic deletion of *Sox9* was also affecting HOPX expression in the adult DG at 3 months of age. Both *Sox9^fl/fl^;Wnt3a^iresCre/+^* and *Sox9^fl/fl^;Nestin‐Cre* mutants usually die shortly after birth probably due to loss of *Sox9* in tissues outside the CNS; therefore, this analysis was restricted to *Sox9^fl/fl^;Sox1^Cre/+^* mutants and two surviving *Sox9^fl/fl^;Nestin‐Cre* animals. HOPX expression in the SGZ was significantly reduced in the DG of *Sox9^fl/fl^;Sox1^Cre/+^* mice compared to controls, while only partially reduced in *Sox9^fl/fl^;Nestin‐Cre* mice (Figure 6a,b). Because of the restricted number of samples, statistical analysis was not possible in this sample group. Nevertheless, these data clearly show HOPX expression in the adult DG does not recover upon embryonic *Sox9* deletion.

SOX9 is required for NSCs formation (Scott et al., 2010); therefore, we explored the effect of embryonic *Sox9* deletion on adult DG NSC number and proliferative activity (Figure 6c–e). The number of NSCs (identified as average of SGZ SOX2+;GFAP+ cells per image) in the adult DG is similarly reduced in both *Sox9^fl/fl^;Sox1^Cre/+^* and *Sox9^fl/fl^;Nestin‐Cre* mutants (Figure 6d). The number of IPs (identified as average SGZ SOX2+;GFAP– cells per image) also appears reduced; however, this was not statistically significant (Figure 6d). We then analyzed proliferation via co‐expression with Ki67. The proportion of active NSCs in *Sox9^fl/fl^;Sox1^Cre/+^* mutants was dramatically reduced compared to controls, indicating the few remaining adult DG NSCs are rarely dividing and mostly quiescent (Figure 6e), while in *Sox9^fl/fl^;Nestin‐Cre* mutants, this proportion was similar to that found in controls (Figure 6e). Similarly, IP proliferation in *Sox9^fl/fl^;Sox1^Cre/+^* mutants was significantly reduced, while only halved in *Sox9^fl/fl^;Nestin‐Cre* mutants compared to controls (Figure 6e). Together these results show the total number of NSCs and IPs are similarly reduced in both *Sox9* mutants, while the proliferation or proportion of active NSCs and IPs appears more affected in *Sox9^fl/fl^;Sox1^Cre/+^* compared to *Sox9^fl/fl^;Nestin‐Cre* mutants. Despite having only two adult *Sox9^fl/fl^;Nestin‐Cre* mutant samples (due to absence of *Sox9* in other tissues compromising perinatal survival), and the need to have more to draw solid conclusions, the tendency toward a reduced severity of the phenotype in these latter mutants compared to *Sox9^fl/fl^;Sox1^Cre/+^* could correlate with the slightly delayed *Sox9* deletion performed by *Nestin‐Cre* compared to *Sox1^Cre^* in the DNE. We speculate that early expression of SOX9 in this region is necessary and also sufficient (in this cellular context) to initiate some of the events underlying expansion of adult DG NSCs, also confirming the regional origin of these cells. However, further analyses are necessary to confirm this hypothesis. In conclusion, these analyses show that expression of HOPX and the formation and expansion of adult NSCs within the DG very likely depend on DNE‐specific expression of SOX9 during embryonic development.

## 4 DISCUSSION

SOX9 is a major factor in NSC formation (Scott et al., 2010) and it is crucial for proper DG development through induction of astrogenesis in the CH (Caramello et al., 2021; Kang et al., 2012). The embryonic origin and molecular mechanisms underlying adult DG NSC formation are still being investigated (Berg et al., 2019; Li et al., 2013; Nicola et al., 2015). To explore the role of SOX9 in this context and potentially clarify the origin of adult DG NSCs, we performed bulk transcriptomic analysis on a combination of complementary conditional deletions of *Sox9* in the developing ARK and identified *Hopx* as being significantly downregulated in the CH in absence of *Sox9*. We explored its expression in the developing ARK and observed distinct differences in levels between the DNE and the adjacent CH, which also correlate with SOX9 expression, suggesting HOPX is a direct or indirect downstream target of SOX9. Despite both factors being markers of adult DG NSCs and highly expressed in CH, we show by performing in vivo lineage tracing and in vitro neurosphere assays that the embryonic CH does not have stem cell potential and nor does it give rise to adult DG NSCs; it is most likely exclusively committed to astrocytic differentiation.

We identified *Hopx* as a gene regulated by SOX9 in the CH, by comparing the transcriptome of archicortices fully (*Sox9^fl/fl^;Sox1^Cre/+^*) and partially (*Sox9^fl/fl^;Nestin‐Cre*) deleted for *Sox9* (Figure 1b). This approach allowed us to overcome the downsides of bulk transcriptomic analysis where spatial information is lost, while keeping its advantages such as deeper sequencing, as seen with analysis of *Sox9* exons (Figure S1). Subsequent in vivo validation of HOPX expression proved the efficacy of our approach, but also showed that weak SOX9‐dependent expression of HOPX is present in the DNE, although at lower levels compared to CH/FE. In addition to *Hopx*, we identified other interesting pathways in which SOX9 might contribute during DG development (Figure 1d), such as regulation of neuronal versus astrocytic differentiation (Kang et al., 2012; Scott et al., 2010; Stolt et al., 2003) and induction of NSC potential via NOTCH and Wnt signaling (Leung et al., 2016; Liu et al., 2013). Of particular interest is the regulation by SOX9 of genes involved in cell adhesion and migration (*Limk2*, *Arhgap35*, *Fn1*, *Myh14*), which are usually correlated with EMT, notably during neural crest cells development (Leung et al., 2016; Lincoln et al., 2007; Liu et al., 2013). These might be similarly involved in neuronal progenitor migration, which we previously observed to be defective in the developing DG lacking *Sox9* (Caramello et al., 2021) and require further investigation.

Our data show a clear difference in expression levels of both SOX9 and HOPX between the DNE and the CH. This correlates with different cell fates: DNE will give rise to DG neurons and NSCs, while CH will form a glial scaffold (Barry et al., 2008; Caramello et al., 2021), as illustrated here by our in vivo and in vitro data. Both SOX9 and HOPX are required, in different contexts, for NSC formation (Berg et al., 2019; Scott et al., 2010) and gliogenesis (Kang et al., 2012; Mizrak et al., 2019; Morel et al., 2017; Zhong et al., 2020; Zweifel et al., 2018). We hypothesize that SOX9, acting upstream of HOPX, might regulate different cell fates in a dose‐dependent manner: at higher levels SOX9 and HOPX may promote astrocytic differentiation in the CH, while at lower levels they induce quiescent NSC commitment in the DNE (Berg et al., 2019). In agreement with this hypothesis, HOPX expression becomes higher in the fimbrial epithelium at later stages of embryonic development as astrocytic differentiation proceeds in this region. This is in line with previous observations of increasing levels of *Hopx* expression at the same time as other astrocytic differentiating genes, such as *Sox9*, *Nfia*, and *Nfib*, during spinal cord development (Kang et al., 2012; Sagner et al., 2021). The clear differences in HOPX and SOX9 expression levels observed between CH and DNE might be due to the CH‐specific morphogens WNT and BMP (Subramanian et al., 2009), which are known to regulate SOX9 expression (Liu et al., 2013), and have also been shown to induce HOPX in cardiac progenitors (Jain et al., 2015). Nevertheless, HOPX expression is dependent on SOX9 in both regions (Figure 3c,d) as previously observed in the developing spinal cord, where deletion of *Sox9* results in reduction in expression of *Hopx* (HOD‐1) (Kang et al., 2012). Whether this regulation is direct or indirect is still unknown and further investigation, such as ATAC‐Seq and/or ChIPseq analysis, or targeted mutation with CRISPR/CAS9 technology, is required to test the functionality of the SOX9 predicted TBSs we identified.

We also show here that *Hopx* expression and expansion of HOPX+ cells rely on DNE‐specific SOX9 expression, both in the developing and adult DG. This correlates with lower numbers and proliferation rates of NCSs and IPs in the adult DG of both types of *Sox9* mutant mice analyzed here, in agreement with HOPX being required for NSC formation (Berg et al., 2019; Li et al., 2015). However, HOPX expression and NSC and IP proliferation are less affected in the adult DG of *Sox9^fl/fl^;Nestin‐Cre* compared to *Sox9^fl/fl^;Sox1^Cre/+^* mutants. This may be because SOX9 is transiently expressed in the early DNE of *Nestin‐Cre* mutants, which may be sufficient to initialize molecular programs to specify NSC fate or regulate their expansion early postnatally (Nicola et al., 2015). This analysis is based on the only two viable adult *Sox9^fl/fl^;Sox1^Cre/+^* mice; therefore, more samples are needed to confirm these data. Nevertheless, this possibility is also reflected by the fact that reduction of *Notch1* expression, known to regulate NSC quiescence (Urbán et al., 2019), in E13.5 *Sox9^fl/fl^;Nestin‐Cre* ARK is less severe compared to *Sox9^fl/fl^;Sox1^Cre/+^*. This all suggests a dose‐dependent role for SOX9 and HOPX in NSC fate commitment or proliferation in this context too.

In conclusion, we report here the identification of *Hopx* as a potential new downstream target of SOX9 during DG development, with which it might cooperate toward both astrocytic and NSCs commitment, potentially in a dosage‐dependent manner. Moreover, we show that high expression of SOX9 and HOPX in the CH does not lead to formation of DG NSCs, despite both being markers of NSCs. The specific pathways or molecular mechanisms preventing the CH from generating NSCs are unknown, but they are likely to be intrinsic to the cells given their lack of NSC potential in vitro and in vivo. Finally, because CH is the first region in the developing brain to give rise to neurons, the CR cells (Gu et al., 2011), it may also represent the first region to undergo the gliogenic switch. This may explain the upregulation of astrocytic differentiation inducing factors in this domain, at this particular stage. Presumably, given the importance of CR cells for patterning subsequent cortical development (Meyer et al., 2019), it is critical to prevent their continued generation and for their precursors to lose stem cell potential earlier and independently of the remaining neuroepithelium. This further suggests a cell intrinsic mechanism, such as number of cell divisions, built into the process of switching (Gao et al., 2014). The DNE/CH may thus represent an interesting paradigm to investigate the temporal regulation of the gliogenic switch.

## CONFLICT OF INTEREST

The authors declare no conflict of interest.

## Supporting information

Figure S.1 **Bulk RNAseq validation analyses**. **(A, B)** PCA plots of *Sox9^fl/fl^;Sox1^Cre/+^* and *Sox9^fl/fl^;Nestin‐Cre* samples compared to their controls. We observe a greater variance in the transcriptome of *Sox9^fl/fl^;Sox1^Cre/+^* samples compared to their controls related to their genotype (**A**), in contrast with *Sox9^fl/fl^;Nestin‐Cre* samples (**B**). **(C)** Validation of RNAseq quality was also assessed by analysis of *Sox9* exons expression in mutants versus control embryos. In *Sox9* conditional allele, only *Exon2* and *Exon3* (orange boxes) are lost upon Cre recombination, while *Exon1* (yellow box) is still present (blue triangles indicate loxP sites). In our RNAseq, normalized counts of *Sox9* exon reads show *Exon1* is expressed in both controls and *Sox9* mutants, while *Exon2* and *Exon3* are reduced in *Sox9* mutants compared to controls, confirming samples genotype and sequencing quality. PCA: principal component analysis.

Figure S.2 **Analysis on UCSC website of SOX9 transcription binding sites within *Hopx* promoter region. (A)** Schematic of *Hopx* transcripts showing the 4 coding exons and direction of transription. We focused the analysis shown in (**B**) exclusively in the region here highlighted in pink. **(B)** Four predicted SOX9 transcription binding sites (TBSs), both from JASPAR downloaded matrices (under “Supplied User Track”) and JASPAR CORE 2022 database, are found in the regulatory region of *Hopx*, 5 kb upstream transcription starting site. Three of these (yellow highlight) are found in the *Hopx*‐specific enhancer e27364, and one (light blue highlight) in a region permissive to transcription from E15.5 in mouse (Gorkin et al., 2020). **(C)** Colour legend of chromatin state.

Figure S.3 **Proliferation in the LEF1‐ CH is not affected in absence of *Sox9* at E13.5. (A,B)** Double immunostaining for Ki67 and LEF1 (**A**) performed on E13.5 *Sox9^fl/fl^* control and *Sox9^fl/fl^;Sox1^Cre/+^*, *Sox9^fl/fl^;Nestin‐Cre* and *Sox9^fl/fl^;Wnt3a^iresCre/+^* mutant embryos. (**B**) No statistically significant difference in number of Ki67+ cells (normalised for CH pixel area) was found in the CH (LEF1‐ area) of all *Sox9* mutants compared to controls. A schematic representation of the ARK analysed in this figure is shown in Fig. 2.B. DNE: dentate neuroepithelium; CH: cortical hem. Scale bars represent 50 μm.

Figure S.4 **Proliferation of HOPX+ cells in the E18.5 forming DG is specifically affected when *Sox9* is deleted in the DNE. (A,B)** Double immunostaining of Ki67 and HOPX in the forming DG of E18.5 *Sox9^fl/fl^* control and *Sox9^fl/fl^;Sox1^Cre/+^*, *Sox9^fl/fl^;Nestin‐Cre* and *Sox9^fl/fl^;Wnt3a^iresCre/+^* mutant embryos (**A**). Proliferation of HOPX+ cells (**B**) is significantly reduced in *Sox9^fl/fl^;Sox1^Cre/+^* (45.60 ± 3.08%, P = 0.003) and *Sox9^fl/fl^;Nestin‐Cre* mutants (46.83% ± 2.59%, P = 0.0065) compared to controls (56.03% ± 2.16%), while is not affected in *Sox9^fl/fl^;Wnt3a^iresCre/+^* mutants (One‐way ANOVA P = 0.0008), suggesting HOPX+ cell proliferation in the forming DG relies on SOX9 expression in the DNE. A schematic representation of the deveoplin DG analysed in this figure is shown in Fig. 2.C (right). DG: dentate gyrus; DNE: dentate neuroepithelium. Scale bars represent 100 μm.

Figure S.5 **Analysis of differentiated neurospheres from E13.5 dissected archicortices**. Immunostaining of differentiated DIV14 Tomato‐ spheres and Tomato+ aggregates for PDGFRa with GFAP (**A**) and PROX1 with REELIN (**B**) together with endogenous Tomato to analyse cell differentiation towards glial and neuronal fates. Double positive cells are indicated with the yellow arrowhead. Differentiation was quantified as total expression of each indicated marker (**C**) and proportion of each cell type originating from Tomato+ cells (**D**). DIV: days in vitro. Scale bar represent 100 μm in (A) and (B).

Figure S.6 **Lineage tracing of *Wnt3a^iresCre^* in the adult DG**. Representative image of endogenous expression of Tomato in the hippocampus of 1 month old *Wnt3a^iresCre/+;^R26^Tomato/+^* mice. DG: dentate gyrus; CA1,2,3: *Cornus Ammonis* 1,2,3; GCL: granule cell layer. Scale bar represent 100 μm.

Table S1

Table S2

Table S3

Table S4

## Data Availability

The RNA dataset that supports the findings of this study is openly available in NCBI's Gene Expression Omnibus and is accessible through GEO Series accession number GSE196386 at https://www.ncbi.nlm.nih.gov/geo/query/acc.cgi?acc=GSE196386.
