# Supplementary figures and images for: The cortical hem lacks stem cell potential despite expressing SOX9 and HOPX
Source: Dev Neurobiol. 2022 Sep 20;82(7-8):565–80. doi: 10.1002/dneu.22899 (PMC9826121; doi:10.1002/dneu.22899)

A

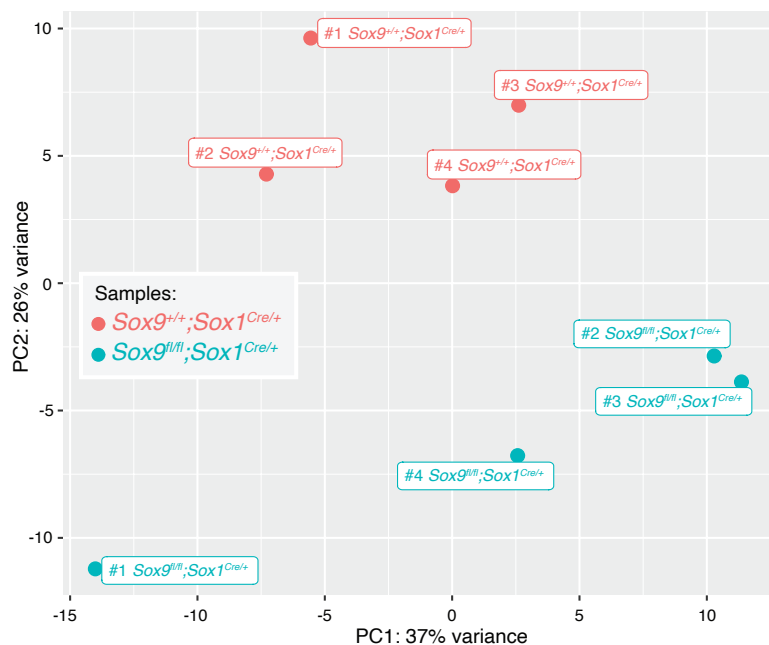

B

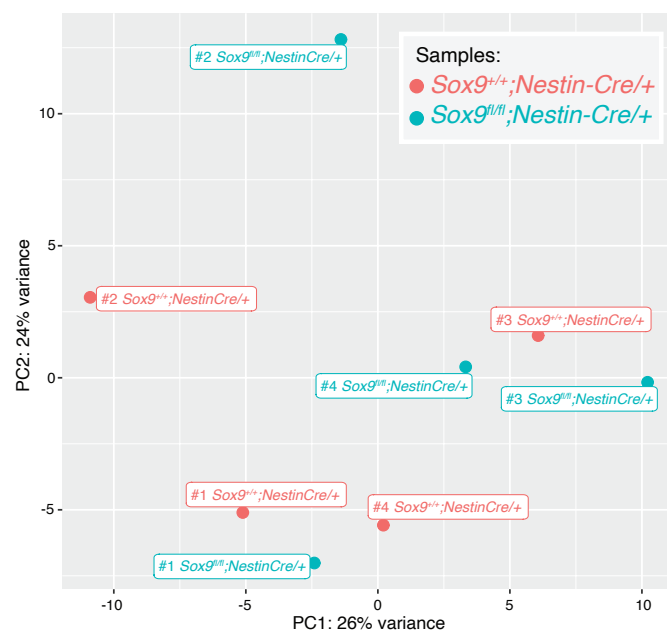

C

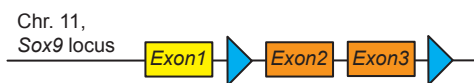

Normalized Exon Counts Sox9

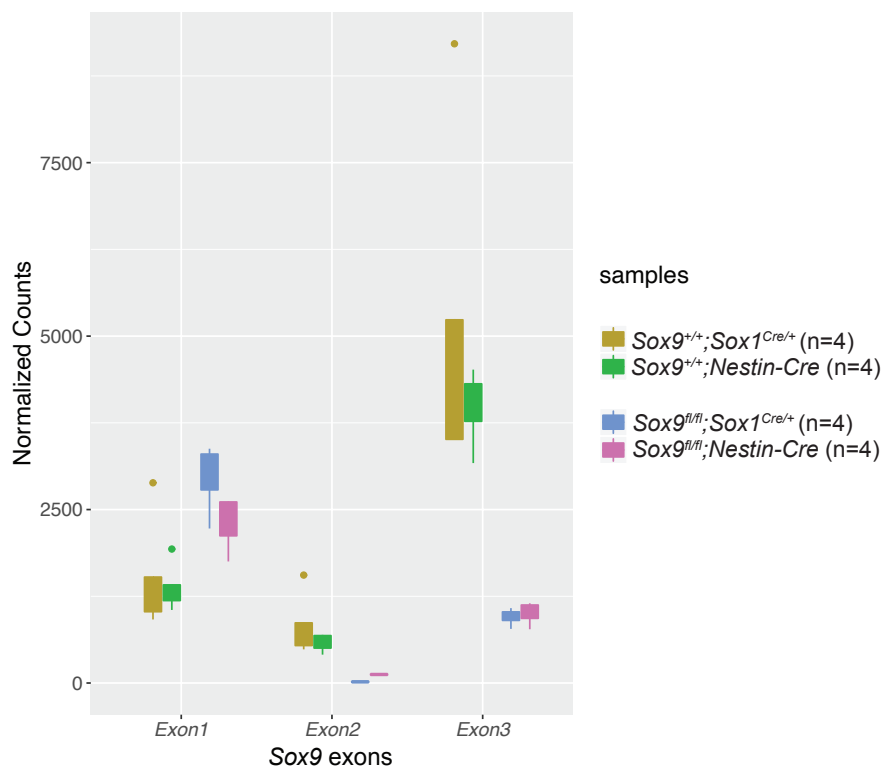

Supplement: Supplementary file 1 — Figure S.1 Bulk RNAseq validation analyses. (A, B) PCA plots of Sox9fl/fl;Sox1Cre/+ and Sox9fl/fl;Nestin‐Cre samples compared to their controls. We observe a greater variance in the transcriptome of Sox9fl/fl;Sox1Cre/+ samples compared to their controls related to their genotype (A), in contrast with Sox9fl/fl;Nestin‐Cre samples (B). (C) Validation of RNAseq quality was also assessed by analysis of Sox9 exons expression in mutants versus control embryos. In Sox9 conditional allele, only Exon2 and Exon3 (orange boxes) are lost upon Cre recombination, while Exon1 (yellow box) is still present (blue triangles indicate loxP sites). In our RNAseq, normalized counts of Sox9 exon reads show Exon1 is expressed in both controls and Sox9 mutants, while Exon2 and Exon3 are reduced in Sox9 mutants compared to controls, confirming samples genotype and sequencing quality. PCA: principal component analysis. [file DNEU-82-565-s001.pdf]

A

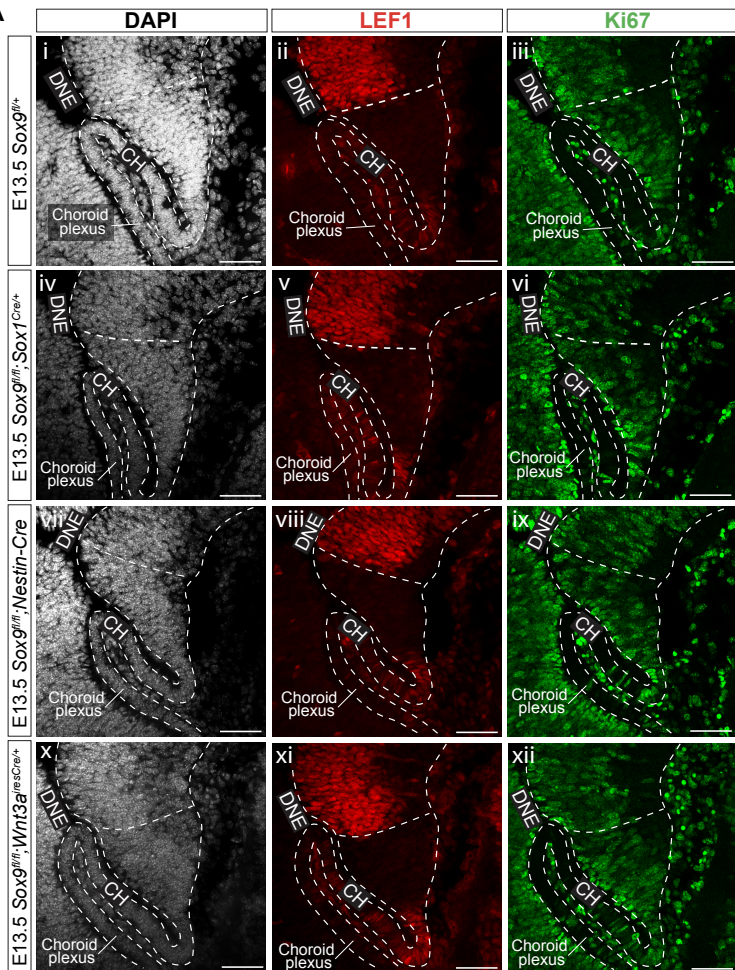

B

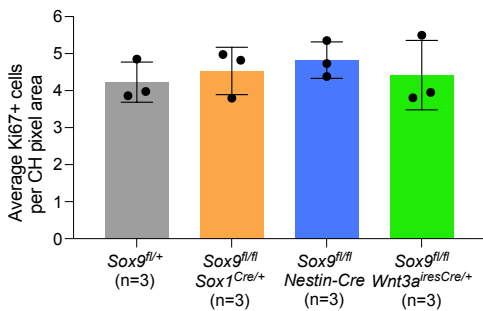

Supplement: Supplementary file 3 — Figure S.3 Proliferation in the LEF1‐ CH is not affected in absence of Sox9 at E13.5. (A,B) Double immunostaining for Ki67 and LEF1 (A) performed on E13.5 Sox9fl/fl control and Sox9fl/fl;Sox1Cre/+ , Sox9fl/fl;Nestin‐Cre and Sox9fl/fl;Wnt3airesCre/+ mutant embryos. (B) No statistically significant difference in number of Ki67+ cells (normalised for CH pixel area) was found in the CH (LEF1‐ area) of all Sox9 mutants compared to controls. A schematic representation of the ARK analysed in this figure is shown in Fig. 2.B. DNE: dentate neuroepithelium; CH: cortical hem. Scale bars represent 50 μm. [file DNEU-82-565-s009.pdf]

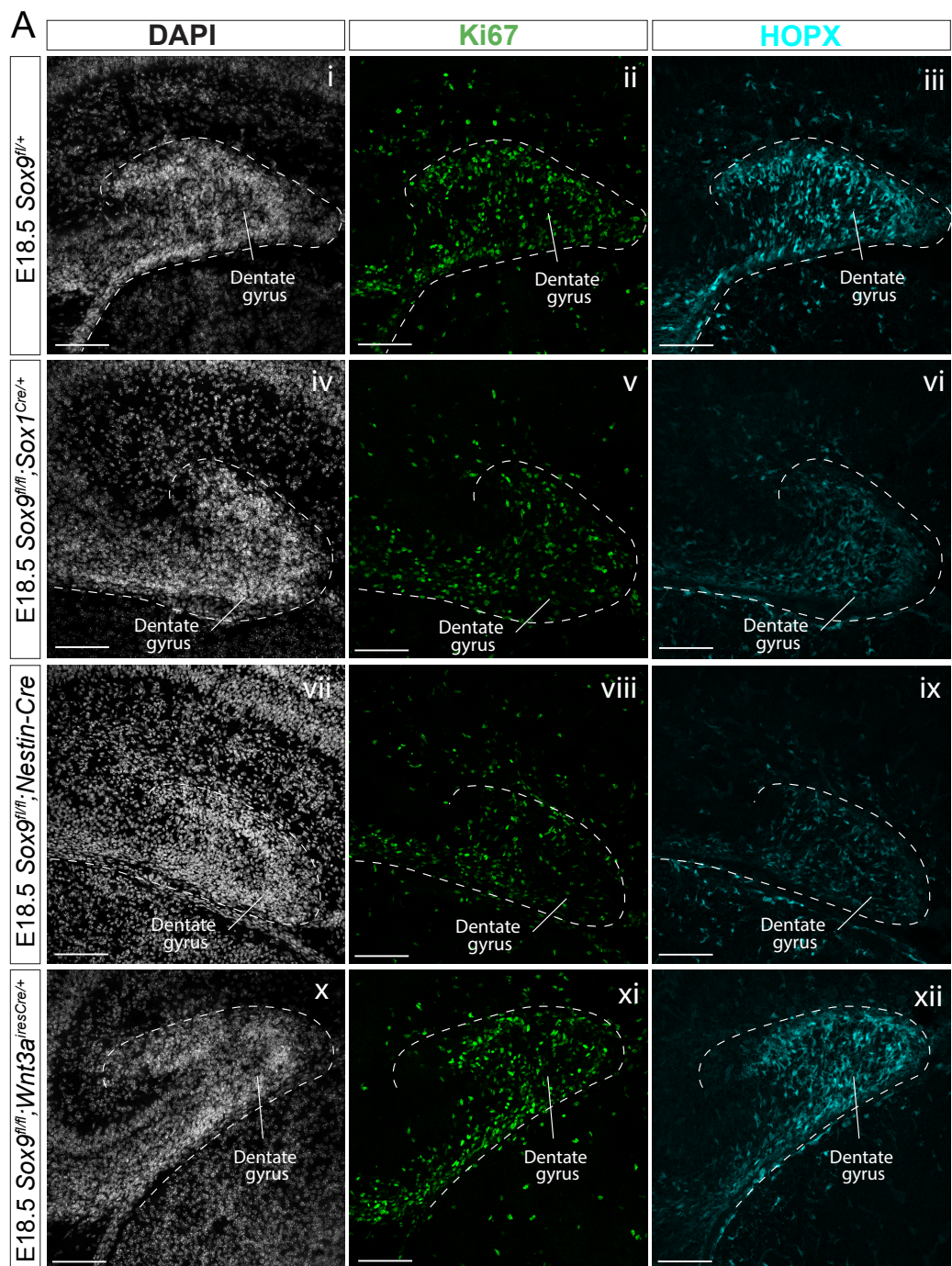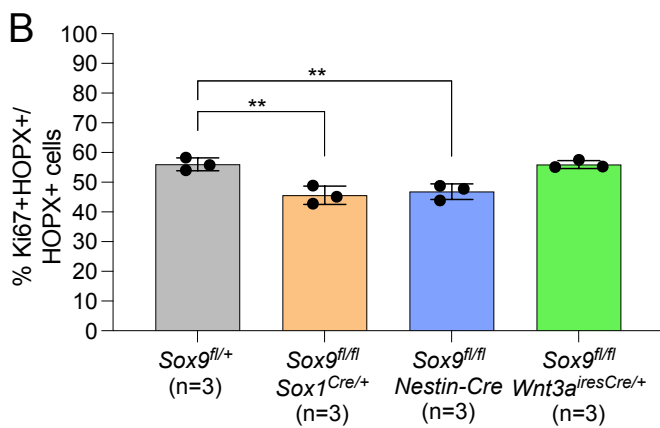

Supplement: Supplementary file 4 — Figure S.4 Proliferation of HOPX+ cells in the E18.5 forming DG is specifically affected when Sox9 is deleted in the DNE. (A,B) Double immunostaining of Ki67 and HOPX in the forming DG of E18.5 Sox9fl/fl control and Sox9fl/fl;Sox1Cre/+ , Sox9fl/fl;Nestin‐Cre and Sox9fl/fl;Wnt3airesCre/+ mutant embryos (A). Proliferation of HOPX+ cells (B) is significantly reduced in Sox9fl/fl;Sox1Cre/+ (45.60 ± 3.08%, P = 0.003) and Sox9fl/fl;Nestin‐Cre mutants (46.83% ± 2.59%, P = 0.0065) compared to controls (56.03% ± 2.16%), while is not affected in Sox9fl/fl;Wnt3airesCre/+ mutants (One‐way ANOVA P = 0.0008), suggesting HOPX+ cell proliferation in the forming DG relies on SOX9 expression in the DNE. A schematic representation of the deveoplin DG analysed in this figure is shown in Fig. 2.C (right). DG: dentate gyrus; DNE: dentate neuroepithelium. Scale bars represent 100 μm. [file DNEU-82-565-s007.pdf]

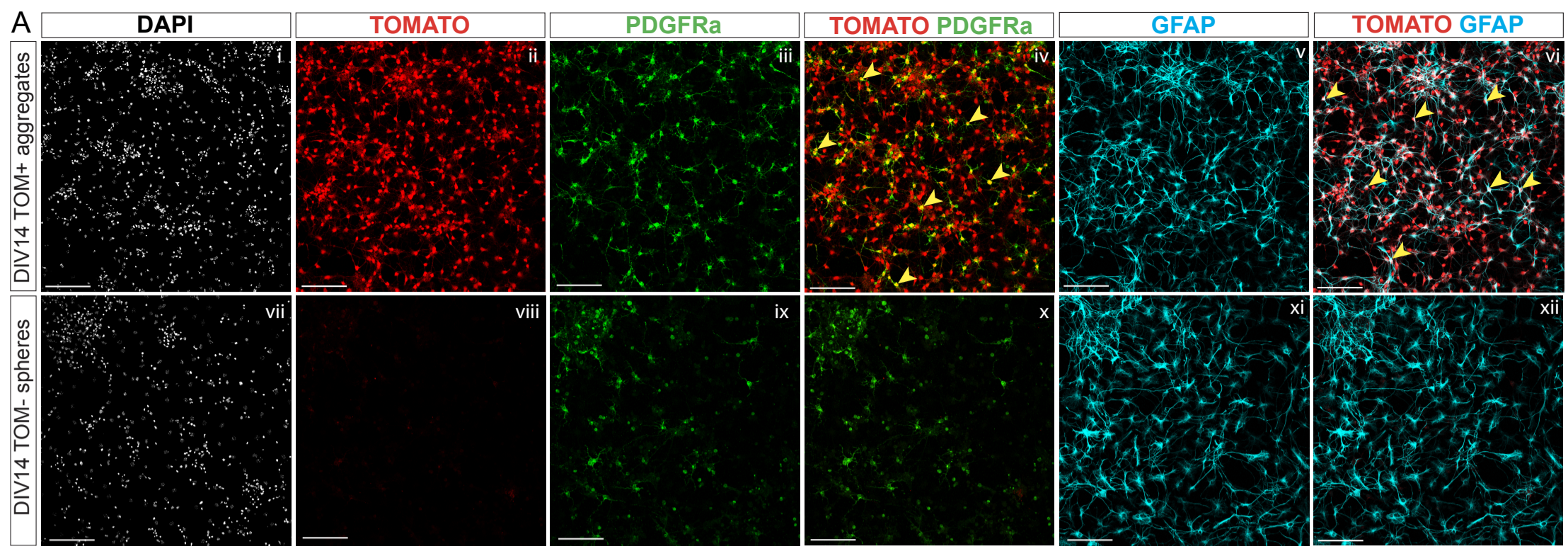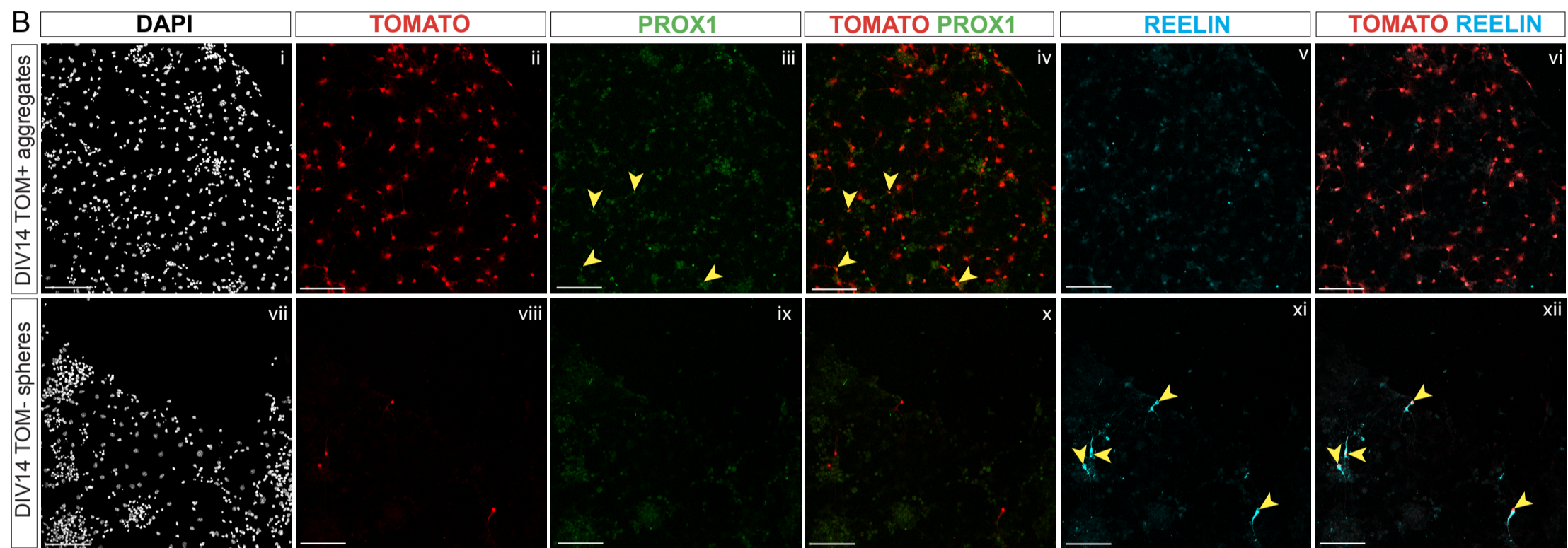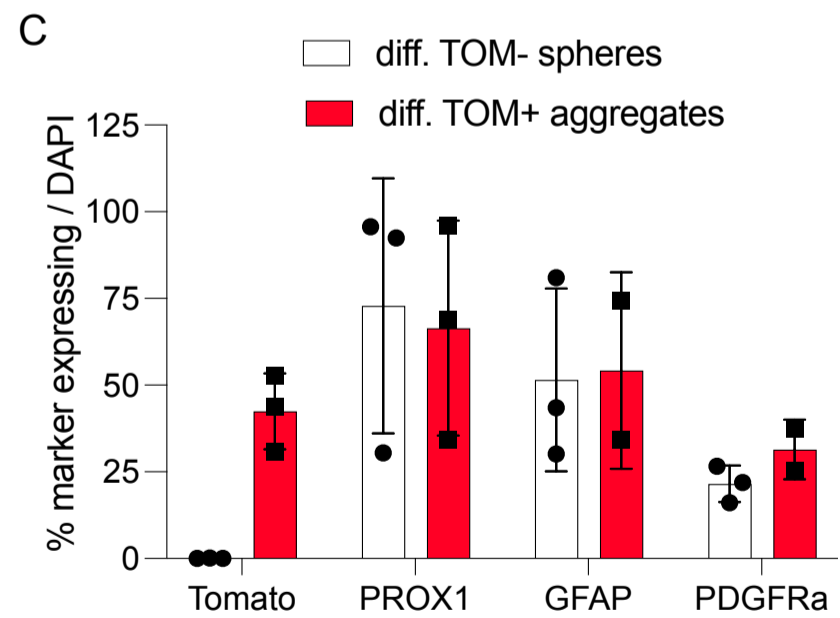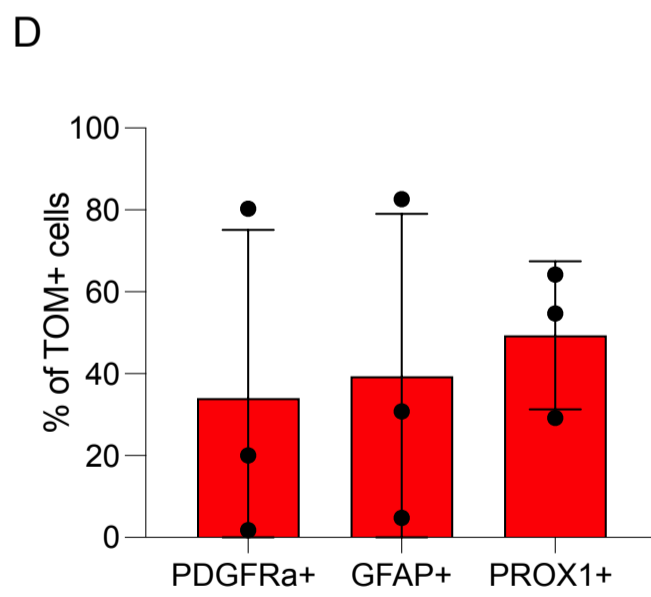

Supplement: Supplementary file 5 — Figure S.5 Analysis of differentiated neurospheres from E13.5 dissected archicortices. Immunostaining of differentiated DIV14 Tomato‐ spheres and Tomato+ aggregates for PDGFRa with GFAP (A) and PROX1 with REELIN (B) together with endogenous Tomato to analyse cell differentiation towards glial and neuronal fates. Double positive cells are indicated with the yellow arrowhead. Differentiation was quantified as total expression of each indicated marker (C) and proportion of each cell type originating from Tomato+ cells (D). DIV: days in vitro. Scale bar represent 100 μm in (A) and (B). [file DNEU-82-565-s006.pdf]

*Wnt3a<sup>iresCre/+</sup>;R26<sup>Tomato/+</sup>*

# TOMATO

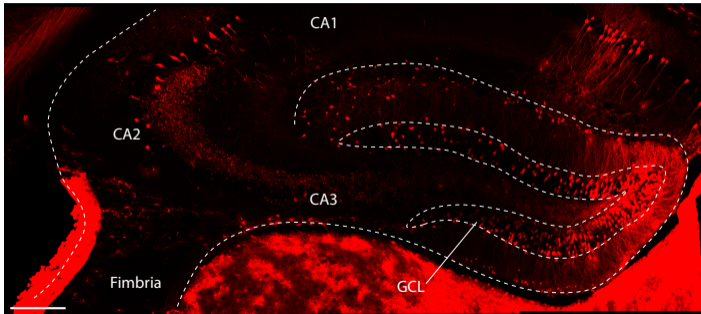

Supplement: Supplementary file 6 — Figure S.6 Lineage tracing of Wnt3airesCre in the adult DG. Representative image of endogenous expression of Tomato in the hippocampus of 1 month old Wnt3airesCre/+;R26Tomato/+ mice. DG: dentate gyrus; CA1,2,3: Cornus Ammonis 1,2,3; GCL: granule cell layer. Scale bar represent 100 μm. [file DNEU-82-565-s003.pdf]
