# Supplementary material for: The cortical hem lacks stem cell potential despite expressing SOX9 and HOPX
Source: Dev Neurobiol. 2022 Sep 20;82(7-8):565–80. doi: 10.1002/dneu.22899 (PMC9826121; doi:10.1002/dneu.22899)
Supplement: Supplementary file 2 — Figure S.2 Analysis on UCSC website of SOX9 transcription binding sites within Hopx promoter region. (A) Schematic of Hopx transcripts showing the 4 coding exons and direction of transription. We focused the analysis shown in (B) exclusively in the region here highlighted in pink. (B) Four predicted SOX9 transcription binding sites (TBSs), both from JASPAR downloaded matrices (under “Supplied User Track”) and JASPAR CORE 2022 database, are found in the regulatory region of Hopx, 5 kb upstream transcription starting site. Three of these (yellow highlight) are found in the Hopx‐specific enhancer e27364, and one (light blue highlight) in a region permissive to transcription from E15.5 in mouse (Gorkin et al., 2020). (C) Colour legend of chromatin state. [file DNEU-82-565-s002.pdf]

A

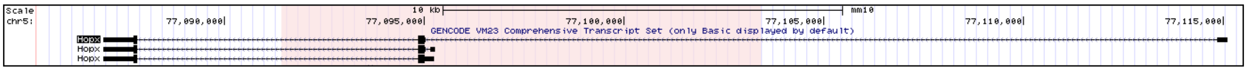

B

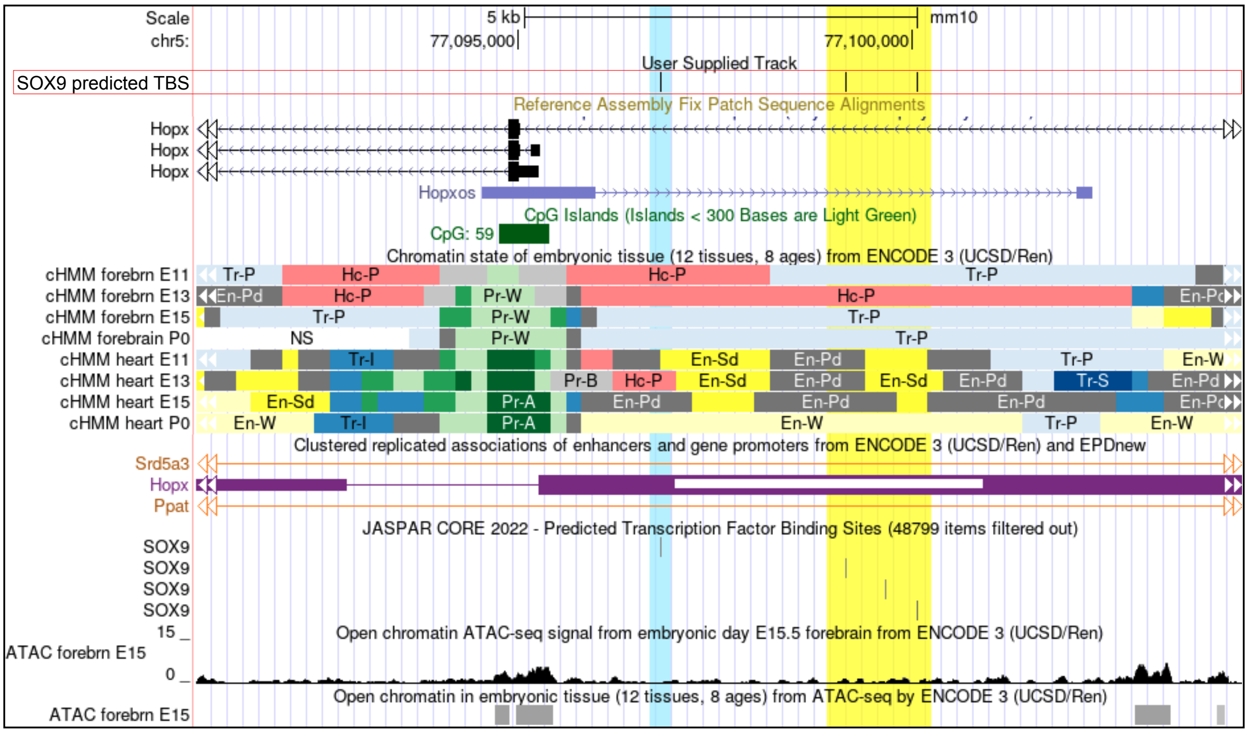

C

- State 1 - Dark Green - Promoter, Active (Pr-A)
- State 2 - Light Green - Promoter, Weak (Pr-W)
- State 3 - Light Grey - Promoter, Bivalent (Pr-B)
- State 4 - Green - Promoter, Flanking Region (Pr-F)
- State 5 - Bright Yellow - Enhancer, Strong TSS-distal (En-Sd)
- State 6 - Bright Yellow - Enhancer, Strong TSS-proximal (En-Sp)
- State 7 - Light Yellow - Enhancer, Weak (En-W)
- State 8 - Dark Grey - Enhancer, Poised TSS-distal (En-Pd)
- State 9 - Dark Grey - Enhancer, Poised TSS-proximal (En-Pp)
- State 10 - Dark Blue - Transcription, Strong (Tr-S)
- State 11 - Royal Blue - Transcription, Permissive (Tr-P)
- State 12 - Light Blue - Transcription, Initiation (Tr-I)
- State 13 - State 13 - Salmon - Heterochromatin, Polycomb-associated (Hc-P)
- State 14 - Pink - Heterochromatin, H3K9me3-associated (Hc-H)
- State 15 - White - No significant signal (Ns)
